# Leaf Cuticular Transpiration Barrier Organization in Tea Tree Under Normal Growth Conditions

**DOI:** 10.3389/fpls.2021.655799

**Published:** 2021-06-30

**Authors:** Mingjie Chen, Yi Zhang, Xiangrui Kong, Zhenghua Du, Huiwen Zhou, Zhaoxi Yu, Jianheng Qin, Changsong Chen

**Affiliations:** ^1^College of Life Sciences, Key Laboratory of Tea Biology of Henan Province, Xinyang Normal University, Xinyang, China; ^2^Tea Research Institute, Fujian Academy of Agricultural Sciences, Fuan, China; ^3^Horticultural Plant Biology and Metabolomics Center, Haixia Institute of Science and Technology, Fujian Agriculture and Forestry University, Fuzhou, China; ^4^The Fujian Research Branch of the National Tea Genetic Improvement Center, Fuzhou, China

**Keywords:** *Camellia sinensis*, cuticular transpiration rate, epicuticular waxes, intracuticular waxes, wax coverage, cuticle thickness, substructure

## Abstract

The cuticle plays a major role in restricting nonstomatal water transpiration in plants. There is therefore a long-standing interest to understand the structure and function of the plant cuticle. Although many efforts have been devoted, it remains controversial to what degree the various cuticular parameters contribute to the water transpiration barrier. In this study, eight tea germplasms were grown under normal conditions; cuticle thickness, wax coverage, and compositions were analyzed from the epicuticular waxes and the intracuticular waxes of both leaf surfaces. The cuticular transpiration rates were measured from the individual leaf surface as well as the intracuticular wax layer. Epicuticular wax resistances were also calculated from both leaf surfaces. The correlation analysis between the cuticular transpiration rates (or resistances) and various cuticle parameters was conducted. We found that the abaxial cuticular transpiration rates accounted for 64–78% of total cuticular transpiration and were the dominant factor in the variations for the total cuticular transpiration. On the adaxial surface, the major cuticular transpiration barrier was located on the intracuticular waxes; however, on the abaxial surface, the major cuticular transpiration barrier was located on the epicuticular waxes. Cuticle thickness was not a factor affecting cuticular transpiration. However, the abaxial epicuticular wax coverage was found to be significantly and positively correlated with the abaxial epicuticular resistance. Correlation analysis suggested that the very-long-chain aliphatic compounds and glycol esters play major roles in the cuticular transpiration barrier in tea trees grown under normal conditions. Our results provided novel insights about the complex structure–functional relationships in the tea cuticle.

## Introduction

The plant cuticle is an extracellular hydrophobic layer covering the outer epidermal surface of leaves and fruits and protects plants against biotic and abiotic stresses (Eigenbrode and Espelie, 1995; Krauss et al., 1997; Neinhuis and Barthlott, 1997; Yeats and Rose, 2013; Silva et al., 2017). The primary function of the cuticle is to regulate nonstomatal water loss, thus facilitating the adaption of plants to a changing environment (Riederer and Schneider, 2001). The cuticle is composed principally of cutin polyester polymer and soluble cuticular waxes and also contains polysaccharides and proteins (Fernández et al., 2017). The cutin mainly consists of C16 and C18 hydroxyl fatty acids that are cross-linked by ester bonds to form a stable polymer matrix (Nawrath, 2006; Pollard et al., 2008; Fich et al., 2016). Cuticular waxes are mixtures of very-long-chain aliphatic compounds (acids, alkanes, aldehydes, ketones, alcohols, and esters) and cyclic compounds (triterpenoids, tocopherols, and sterols; Samuels et al., 2008). Part of the cuticular waxes is filled into the cutin framework as intracuticular waxes, and part of the waxes is deposited on the surface of the cutin matrix as epicuticular waxes (Jeffree, 2006). Previous studies have reported that the cyclic compounds are mainly located in intracuticular waxes. In contrast, the very-long-chain aliphatic compounds are widely distributed in the epicuticular and intracuticular waxes (Weissflog et al., 2010; Jetter and Riederer, 2016; Zeisler and Schreiber, 2016; Lino et al., 2020; Zhang et al., 2020). Cuticular wax components differ among plant species, developmental stages, tissue types, and even between both sides of the same leaf (Jetter and Riederer, 2016; Zhu et al., 2018; Cheng et al., 2019; Romero and Rose, 2019; Diarte et al., 2020; Lino et al., 2020; Zhang et al., 2020).

There is a long-standing interest to understand the structure–function relationships of the cuticle. Previous studies established that the cuticular transpiration barriers are mainly constituted by cuticular waxes rather than the cutin polymer matrix (Riederer and Schreiber, 1995; Richardson et al., 2007). The cuticular transpiration barrier is mainly contributed by aliphatic compounds, which are located on the intracuticular waxes (Jetter and Riederer, 2016; Zeisler-Diehl et al., 2018; Cheng et al., 2019; Zhang et al., 2020). Recently, there were controversial reports suggesting that cyclic compounds are negatively correlated with cuticular transpiration rates of developing fruits or under drought stress (Schuster et al., 2016; Romero and Rose, 2019; Lino et al., 2020; Zhang et al., 2020); however, other studies found that the cyclic compounds do not contribute to the transpiration barrier or even are positively correlated with the cuticular transpiration rate (Buschhaus and Jetter, 2012; Jetter and Riederer, 2016; Staiger et al., 2019).

Jetter and Riederer (2016) chose eight different plant species of which cuticles belong to two major groups based on the presence and contents of alicyclic compounds of the adaxial leaf surface. Correlation analysis suggests that cuticular transpiration resistance is associated with aliphatic compounds. By now, there are a few studies about the abaxial cuticle, but it remains unclear whether the abaxial cuticle possesses a similar structural arrangement as the adaxial surface. In addition, most previous studies only concentrated on one species or multiple different plant species under the assumption that they may share similar cuticular characteristics. Few studies focus on multiple germplasms of the same plant species, which has the advantage to exploit the variations to boost the power of correlation analysis. In this study, eight different tea germplasms were selected, their cuticles essentially belong to the same type (in terms of the contents and compositions of VLCFA derivatives and alicyclic compounds), but various degrees of variations in cuticular parameters were found. The wax compositions from the adaxial and the abaxial leaf surfaces were analyzed, along with the cuticular transpiration rates (or resistance) from cuticle substructures. This study is trying to address the following questions: (1) To what degree the abaxial cuticular transpiration contributes to the total leaf cuticular transpiration? (2) How the transpiration barrier is organized on the abaxial surface? (3) Which are the wax compounds that contribute to the cuticular transpiration barriers at individual cuticle substructure?

## Materials and Methods

Eight *Camellia sinensis* germplasms, namely, *Jinguanyin*, *0316B*, *Wuniuzao*, *0306A*, *0306H*, *Fuyun 20*, *0202-10*, and *Hongyafoshou*, were clonally propagated and planted in the tea garden at the Tea Research Institute of Fujian Academy of Agricultural Sciences (Fuan, China; 119.3° E, 27.1° N). They were managed by the regular agricultural practice. In May 2019, they had been growing for 12 years since planting. When the growing twigs reached the stage of one bud and seven leaves, the fifth leaf was used for the investigation.

### Transmission Electron Microscopy

For transmission electron microscopy (TEM) analysis, the sample was prepared in accordance with the method described in the study by Zhu et al. (2018). The central part of the fifth leaf was cut into small pieces (2 mm × 4 mm) and fixed in 5% (v/v) glutaraldehyde solution overnight in a freezer at 4°C. Samples were rinsed with 0.1 M PBS buffer (pH 7.2), post-fixed in 1% (w/v) osmium tetroxide, then dehydrated through 30% (v/v) and 50% (v/v) ethanol. Samples were stained with saturated uranyl acetate in 70% (v/v) ethanol overnight, and then rinsed with 70% (v/v) ethanol several times to remove unbound dye. Samples were dehydrated in 90% (v/v) and 100% ethanol; then the ratio of acetone to ethanol was increased in sequential treatments. Samples were embedded in a graded acetone/Epon/Spurr’s epoxy resin and polymerized, then sectioned, and observed under a TEM (HT7700, Hitachi, Japan).

### Scanning Electron Microscopy and Stomata Parameter Measurement

The fifth leaf was collected from the eight germplasms. Samples were air-dried at room temperature. Before scanning electron microscopy (SEM) observation, small pieces of samples were fixed to aluminum sample holders, freeze-dried (HCP-2 critical point dryer, Hitachi, Japan), sputtered (IB5 ion coater, Eiko, Japan) with a thin layer of gold, then observed under a SEM (JEM-6380LV, JEOL, Japan). Leaf stomatal density was determined from 10 SEM images with different fields of view. Guard cell length and guard cell pair width were calculated by using the ImageJ software.

### Cuticle Thickness Measurement

The cuticle thickness was measured from TEM images. Three biological replicates were used. For each biological replicate, at least six measurements were taken from different cuticle positions, and the results were expressed as average ± SE.

### Wax Sampling

The epicuticular waxes were isolated by the method described in the study by Zhang et al. (2020). Delipidated gum arabic in 90% (w/w) aqueous solution was evenly applied to the leaf surface by a soft paintbrush; dry polymer film was peeled off and collected into a glass tube containing 21 ml of chloroform: water (2:1, v/v) and 75 μg of internal standard n-tetracosane (Sigma-Aldrich, St. Louis, United States). After vigorous vortexing and phase separation, the organic phase was transferred into a new glass tube. The extraction was repeated once, and the organic phases were combined and evaporated under the CentriVap Console (Labconco, KS, United States) to obtain epicuticular waxes. The adaxial epicuticular waxes were isolated first, followed by the isolation of abaxial epicuticular waxes.

After the removal of epicuticular waxes, the leaves were used to extract the intracuticular waxes by rinsing with chloroform (Zhang et al., 2020). The adaxial intracuticular waxes were rinsed first, followed by rinsing with the abaxial intracuticular waxes. The collected chloroform solution was evaporated dry to get the adaxial or the abaxial intracuticular waxes, respectively.

### Wax Analysis

Before gas chromatography–mass spectrometry (GC–MS) and GC-flame ionization detector (FID) analysis, wax samples were derivatized by *N,O*-bis(trimethylsilyl)-trifluoroacetamide (BSTFA, Aldrich, GC grade) containing 1% trimethylchlorosilane (Aldrich) in pyridine (Aldrich, 99.8%, anhydrous). Individual wax components were identified from MS data by comparing their mass spectra with the National Institute of Standard Database (NIST 14). Waxes were quantified from the FID data by normalizing peak area to the internal standard. DB-1 column (30 m × 0.25 mm × 0.25 μm, Agilent, CA, United States) was used. The constant flow rates of helium carrier gas for GC–MS and GC-FID were 1.2 and 1.7 ml min^−1^, respectively. The flow rates for hydrogen, nitrogen, and zero air were 40, 30, and 400 ml min^−1^, respectively. Oven temperature program was initiated at 70°C, raised by 10°C min^−1^ to 200°C, held for 2 min, then raised by 3°C min^−1^ to 320°C, held for 20 min before return to 70°C. The MS detector setting was as follows: EI-70 eV, ionization source temperature: 230°C.

### Cuticular Transpiration Rate Measurement

The cuticular transpiration rate was measured by following the method described in the study by Zhang et al. (2020). Growing twigs at the stage of one bud and seven leaves were harvested and kept in water overnight under dark. The next day, the abaxial surface of the fifth leaf was sprayed with 50 mM ABA to promote stomata closure. For each germplasm, six different treatments were performed to the fifth leaf, namely, (1) control, no additional treatments were applied (T); (2) the adaxial surface was sealed with vaseline (Ad/Vas); (3) the abaxial surface was sealed with vaseline (Ab/Vas); (4) both leaf surfaces were sealed with vaseline (Ad/Vas::Ab/Vas); (5) the adaxial epicuticular waxes were removed by gum arabic, while the abaxial surface was sealed with vaseline (−Ew_Ad_::Ab/Vas); and (6) the abaxial epicuticular waxes were removed by gum arabic, while the adaxial surface was sealed with vaseline (−Ew_Ab_::Ad/Vas). After completing these pretreatments, the fifth leaf was removed and photographed, and the projected leaf area (A) was calculated by ImageJ software. The initial, water-saturated fresh leaf weight (W_i_) was recorded. Leaves were then placed in a controlled dark room (25°C, 50% humidity), and the leaf weight was recorded hourly by balance; the measurement lasted for 8 h (W_t1, 2…8_). Each treatment group included five leaves representing five biological replicates. The cuticular transpiration rates from the total leaf surface (T_Total_C_), the adaxial surface (T_Ad_), the abaxial surface (T_Ab_C_), the adaxial intracuticular waxes (T_Ad/intra_), and the abaxial intracuticular waxes (T_Ab/intra_C_) were calculated by the formula described before (Zhang et al., 2020). The transpiration rate from the epicuticular waxes cannot be directly measured by the method described in the study by Zhang et al. (2020). After the removal of epicuticular wax, the higher the ratio increase of the intracuticular transpiration rate, the higher the epicuticular resistance would be. By using this relationship, here, we define the epicuticular resistance as the relative ratio increase of the intracuticular transpiration rate after the epicuticular wax removal. Thus, in this study, the cuticular transpiration rate and the resistance represent two different terminologies. The adaxial epicuticular resistance (R_Ad/epi_) was calculated by the formula (I):

(I) $$R_{\mathrm{Ad}/\mathrm{epi}}\;\left(\%\right)=\left[\left(T_{\mathrm{Ad}/\mathrm{intra}}-T_{\mathrm{Ad}}\right)/T_{\mathrm{Ad}/\mathrm{intra}}\right]\times 100$$

T_Ad_ and T_Ad/intra_ represent the adaxial cuticular transpiration rates before and after epicuticular wax removal, respectively;

The abaxial epicuticular resistance (R_Ab/epi_C_) was calculated by the formula (II):

(II) $$R_{\mathrm{Ab}/\mathrm{epi}\_C}\;\left(\%\right)=\left[\left(T_{\mathrm{Ab}/\mathrm{intra}\_C}-T_{\mathrm{Ab}\_C}\right)/T_{\mathrm{Ab}/\mathrm{intra}\_C}\right]\times 100$$

T_Ab_ and T_Ab/intra_C_ represent the abaxial cuticular transpiration rates before and after abaxial epicuticular wax removal, respectively.

It is worth mentioning that Jetter and Riederer (2016) defined resistance as the inverse of permeance and different from the definition here.

### Statistical Analysis

The mean and SE were calculated by ANOVA. Significance was determined by one-way ANOVA based on Duncan’s multiple range tests. Regression analysis between cuticular transpiration rate and wax composition was performed by SPSS (V17.0; SPSS, IBM, Armonk, United States). Bivariate correlations based on Pearson’s correlation (two-tailed) were used to determine the significance of correlations between different parameters.

## Results

### The Cuticle Thickness of the Eight Tea Germplasms

**Figure 1:**
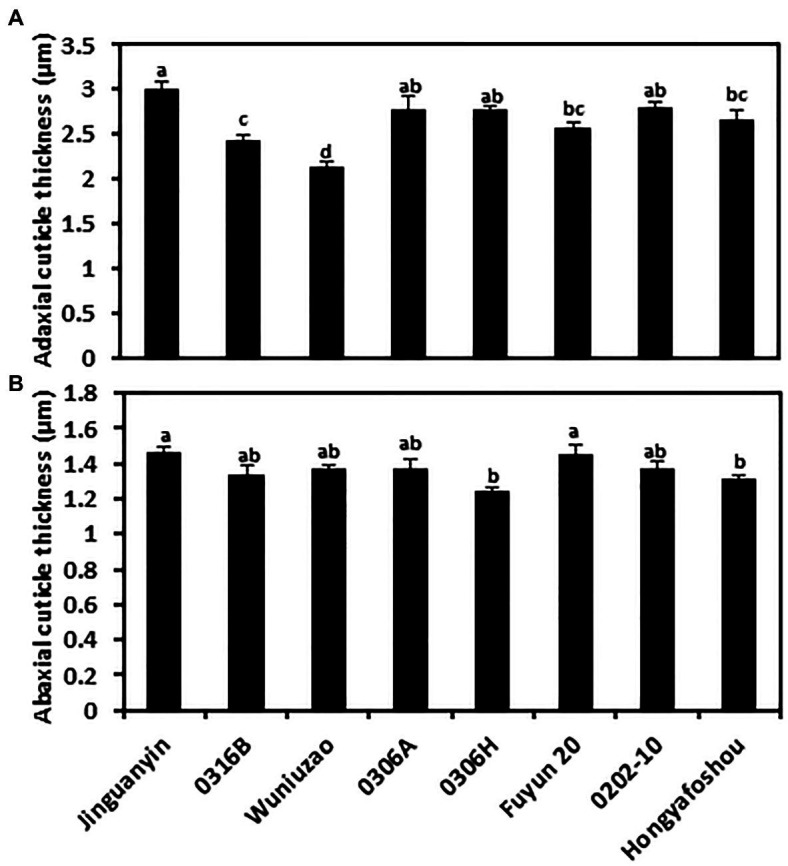
Cuticle thickness from the adaxial and the abaxial cuticle of the eight tea germplasms. **(A)** The adaxial surface; **(B)** the abaxial surface. Different lowercase letters represent the statistical significance (*p* < 0.05).

To measure the cuticle thickness, the fifth leaf of the eight tea germplasms was prepared for TEM observation. Under our sample preparation method, the cuticle showed a whitish appearance, and the cell wall was stained dark (Supplementary Figure S1). Thus, the cuticle was clearly discerned from TEM imaging. Among the eight tea germplasms, the average thickness of the adaxial cuticle ranged from 2.12 to 2.99 μm, with *Wuniuzao* and *Jinguanyin* ranked as the thinnest and the thickest adaxial cuticle, respectively. The abaxial cuticle thickness showed relatively smaller variations and ranged from 1.24 to 1.46 μm, with *0306H* and *Jinguanyin* ranked as the thinnest and the thickest abaxial cuticle, respectively. For individual germplasms, the adaxial cuticle generally was thicker than the abaxial cuticle (Figure 1; Supplementary Figure S1).

### The Epi- and Intracuticular Wax Coverage of the Eight Tea Germplasms

**Figure 2:**
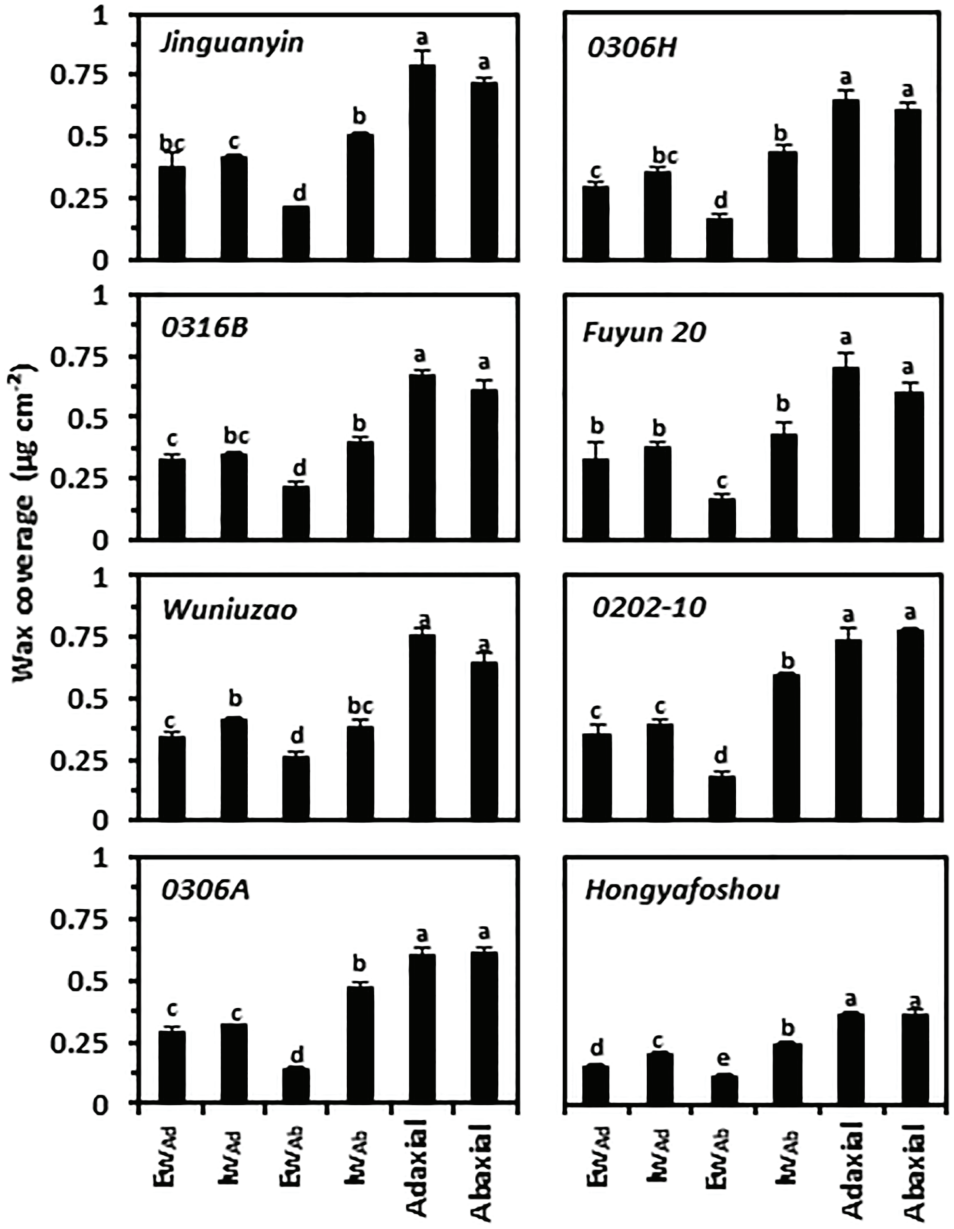
Cuticular wax coverage from the epi- and the intracuticular compartments of both leaf surfaces. Ew: the epicuticular waxes; Iw: the intracuticular waxes; Ad: the adaxial surface; Ab: the abaxial surface. Different lowercase letters represent the statistical significance (*p* < 0.05).

To compare the compositional characteristics of the cuticle from the eight tea germplasms, waxes were isolated from the epi- and intracuticular compartments of both leaf surfaces, and then analyzed by GC–MS and GC-FID. For individual germplasm, the wax coverages from the adaxial and abaxial surfaces did not correlate with their respective cuticle thickness. Although the adaxial cuticle was thicker than the abaxial cuticle (Figure 1; Supplementary Figure S1), the wax coverage from the adaxial surface and the abaxial surface was similar (Figure 2). This suggests that the wax density from the adaxial surface was lower than that of the abaxial surface. In addition, the epicuticular waxes and the intracuticular waxes from the adaxial surface and the abaxial surface showed different wax distribution patterns. On the adaxial surface, the waxes were almost equally distributed between the epicuticular waxes and the intracuticular waxes; in contrast, the intracuticular waxes coverage on the abaxial surface was about 1.45–3.33 times higher than that of the epicuticular waxes. For individual germplasm, the coverage of adaxial the epicuticular waxes generally was higher than that of the abaxial the epicuticular waxes. Among the four cuticular compartments of each germplasm, the coverage of the abaxial intracuticular waxes ranked as the highest except *Wuniuzao* (Figure 2). *Hongyafoshou* showed the lowest total wax coverage (0.73 μg cm^−2^) among the eight tea germplasms. Accordingly, its wax coverage from each cuticular compartment was also the lowest.

### The Epi- and Intracuticular Wax Compositions of the Eight Tea Germplasms

**Figure 3:**
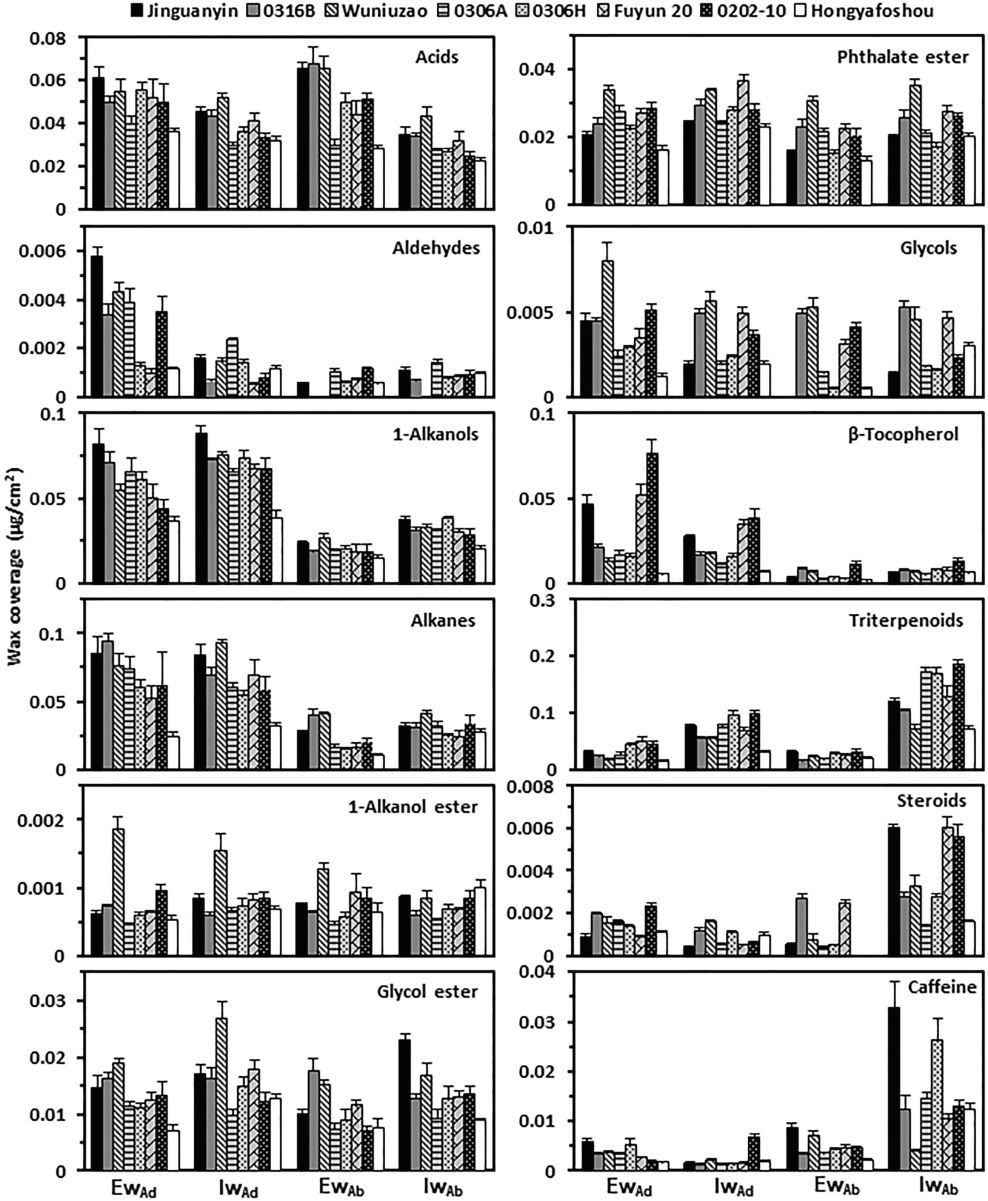
The wax compositional comparison from the epi- and intracuticular compartments among the eight tea germplasms. Ew_Ad_: the adaxial eipcuticular waxes; Iw_Ad_: the adaxial intracuticular waxes; Ew_Ab_: the abaxial eipcuticular waxes; Iw_Ab_: the abaxial intracuticular waxes.

At the chemical class level, tea leaf waxes include glycols, caffeine, VLCFAs, and their primary alcohols, alkyl esters, aldehydes, and alkane derivatives; mature tea leaves also contained considerable amounts of pentacyclic triterpenoids, steroids, and tocopherols (Zhu et al., 2018; Chen et al., 2020; Zhang et al., 2020). The adaxial epicuticular and intracuticular waxes were dominated by VLCFA compounds (acids, aldehydes, 1-alcohols, and alkanes) in all germplasms, which accounted for 46–67% of the total epicuticular wax coverage and 41–54% of the total intracuticular wax coverage (Figure 3). Compared to the abaxial epicuticular waxes, the adaxial epicuticular waxes showed higher coverage of aldehydes, 1-alkanols, alkanes, and β-tocopherol. Compared to the abaxial intracuticular waxes, the adaxial intracuticular waxes showed higher coverage of 1-alkanols, alkanes, and β-tocopherol, but showed lower coverage of triterpenoids, steroids, and caffeine (Figure 3). Due to these biased distributions of individual wax components on both leaf surfaces, the adaxial coverages of 1-alkanols, alkanes, and β-tocopherol were higher than those of the abaxial surface; in contrast, the adaxial coverage of triterpenoids, steroids, and caffeine was lower than that of the abaxial surface.

### The Cuticular Transpiration Rates From Different Leaf Surfaces and Cuticular Compartments

**Figure 4:**
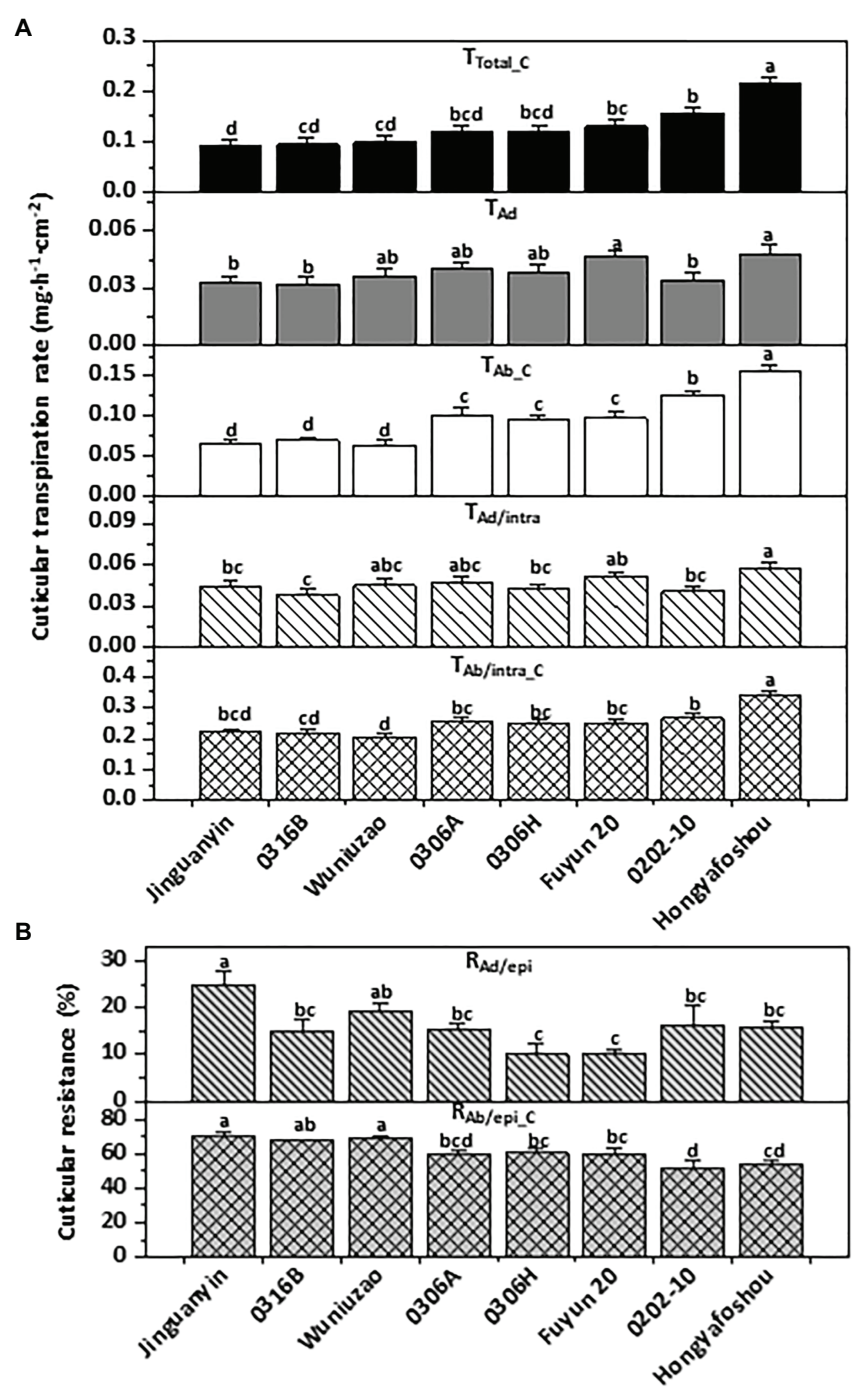
Cuticular transpiration rate **(A)** and epicuticular resistance **(B)** of the eight tea germplasms. Different lowercase letters represent the statistical significance (*p* < 0.05). T_Total_C_: the total cuticular transpiration rate; T_Ad_: the adaxial cuticular transpiration rate; T_Ab_C_: the abaxial cuticular transpiration rate; T_Ad/intra_: the adaxial intracuticular transpiration rate; T_Ab/intra_C_: the abaxial intracuticular transpiration rate; R_Ad/epi_: the adaxial epicuticular resistance; R_Ab/epi_C_: the abaxial epicuticular resistance.

The water transpiration rates from the fifth leaf were measured. On the leaf drying curve, *Hongyafoshou* and *Fuyun 20* reached a constant level 4th h post-excision; however, the remaining six germplasms reached constant levels at the 5th h post-excision (Supplementary Figure S2). The observed leaf transpiration rates for the control and various treatments were recorded; the values at the 1st h and the 5th h post-excision are presented in Supplementary Table S1. The residual stomata transpiration rate was obtained by using the difference in the total transpiration rate between the 1st h and the 5th h of the control leaves (T_1_–T_5_). *Hongyafoshou* and *Fuyun 20* showed higher residual stomata transpiration rates compared to other six tea germplasms. Accordingly, they reached a constant level 1 h earlier than others. The total leaf transpiration rate at the 5th h post-excision was used as proxy of the total cuticular transpiration rate since at this time point the stomata was fully closed. The cuticular transpiration rates from the adaxial surface and the abaxial surface were obtained from the observed data by applying the established formula (Zhang et al., 2020; Figure 4; Supplementary Table S1).

The total cuticular transpiration rate (T_Total_C_) ranged from 0.090 mg.h^−1^.cm^−2^ for *Jinguanyin* to 0.216 mg.h^−1^.cm^−2^ for *Hongyafoshou* (Figure 4A, top panel). Accordingly, *Hongyafoshou* showed higher cuticular transpiration rates from all its cuticular compartments and also lower abaxial epicuticular resistance (R_Ab/epi_C_; Figures 4A,B). The adaxial cuticular transpiration rates showed small variations among these eight tea germplasms; in contrast, the abaxial cuticular transpiration rates showed much larger variations. The abaxial cuticular transpiration rate (T_Ab_C_) was about 1.8–3.3-fold high of the adaxial surface (T_Ad_) in all the eight tea germplasms (Figure 4A). The abaxial intracuticular transpiration rates (T_Ab/intra_C_) were about 4.6–6.5-fold higher than the adaxial intracuticular waxes (T_Ad/intra_). The resistance of the abaxial epicuticular waxes (R_Ab/epi_C_) was about 2.8–6.4-fold higher than that of the adaxial epicuticular waxes (R_Ad/epi_; Figure 4B). Overall, the water loss from the adaxial surface (T_Ad_) accounted for 22–36% of the total leaf water loss (T_Total_C_), while the water loss from the abaxial surface (T_Ab_C_) accounted for 64–78% of the total leaf water loss (Figure 4). It is clear that the adaxial leaf surface showed a better cuticular transpiration barrier compared to its abaxial surface.

Machado et al. (2020) studied 30 different native tree species, including drought deciduous and evergreen, and found that residual stomatal transpiration had a significant impact on the minimum conductance; the stomata distribution pattern in the epidermis was a key factor determining the variation in minimum conductance. To exclude the stomatal effects on the cuticular transpiration rate measurement, in this study the stomata density, guard cell length, and guard cell pair width were measured from SEM images (Supplementary Figure S3A). The average stomata density from these eight tea germplasms ranged from 225 to 240 mm^−2^; no significant difference was observed among them (Supplementary Figure S3B). Significant differences in guard cell length and guard cell pair width were observed among some pairs of germplasm (Supplementary Figures S3C,D). However, no significant correlations were found between these three stomata parameters and the abaxial cuticular transpiration rates. Machado et al. (2020) also observed that for evergreens there is no significant association between stomatal properties and leaf water leaks. Our data suggest that the variations in the abaxial cuticular transpiration rates from these eight tea germplasms likely resulted from other factors rather than stomatal characteristics (Figure 4A; Supplementary Figure S3).

**Figure 5:**
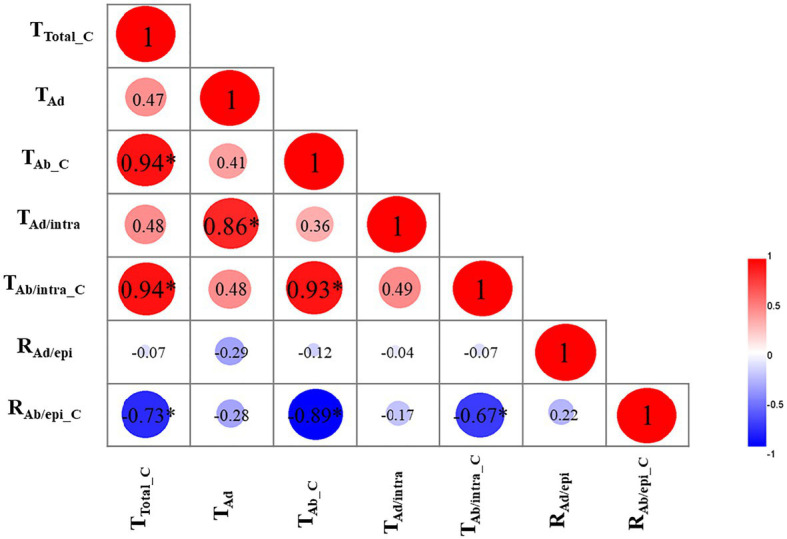
Correlation analysis of cuticular transpiration rate (or resistance; R^2^). T_Total_C_: the total cuticular transpiration rate; T_Ad_: the adaxial cuticular transpiration rate; T_Ab_C_: the abaxial cuticular transpiration rate; T_Ad/intra_: the adaxial intracuticular transpiration rate; T_Ab/intra_C_: the abaxial intracuticular transpiration rate; R_Ad/epi_: the adaxial epicuticular resistance; R_Ab/epi_C_: the abaxial epicuticular resistance. ∗*p* < 0.05.

To uncover the relationships among the cuticular transpiration rates (or resistance), Pearson’s correlation analysis was carried out (Figure 5). The total cuticular transpiration rate (T_Total_C_) was significantly and positively correlated with the abaxial cuticular transpiration rate (T_Ab_C_; *R*^2^ = 0.94) and the abaxial intracuticular transpiration rate (T_Ab/intra_C_; *R*^2^ = 0.94), and significantly and negatively correlated with the abaxial epicuticular resistance (R_Ab/epi_C_; *R*^2^ = −0.73). On the adaxial surface, the adaxial cuticular transpiration rate (T_Ad_) was significantly and positively correlated with the adaxial intracuticular transpiration rate (T_Ad/intra_; *R*^2^ = 0.86); however, T_Ad_ was not correlated with the adaxial epicuticular resistance (R_Ad/epi_). On the abaxial surface, the abaxial cuticular transpiration rate (T_Ab_C_) was significantly and positively correlated with the abaxial intracuticular transpiration rate (T_Ab/intra_C_; *R*^2^ = 0.93) and negatively correlated with the abaxial epicuticular resistance (R_Ab/epi_C_; *R*^2^ = −0.89). The abaxial intracuticular transpiration rate (T_Ab/intra_C_) was significantly and negatively correlated with the abaxial epicuticular resistance (R_Ab/epi_C_; *R*^2^ = −0.67; Figure 5).

### Correlation Analysis Between Cuticular Transpiration Rates and Cuticle Structural Parameters

Pearson’s correlation analyses were carried out to identify the associations of cuticular transpiration rates with wax chemical classes from individual cuticular compartments. We found that the total leaf cuticular transpiration rates (T_Total_C_) were significantly and negatively correlated with the total wax coverage (Ad + Ab; *R*^2^ = −0.56; Table 1). In addition, the abaxial epicuticular resistance (R_Ab/epi_C_) was significantly and positively correlated with the abaxial epicuticular wax coverage (Ab_Ew_; *R*^2^ = +0.56). No significant correlations were found from other cuticular compartments (Table 1).

**Table 1:** Correlation analysis between cuticular transpiration rate (or resistance) and cuticular wax chemical classes (*R*^2^).

	Ad + Ab	Ad_Ew + Iw_	Ab_Ew + Iw_	Ad_Iw_	Ab_Iw_	Ad_Ew_	Ab_Ew_
Acids	−0.62^∗^	−0.29	−0.77^∗^	−0.09	−0.70^∗^	+0.08	+0.57^∗^
Aldehydes	−0.17	−0.42	+0.28	+0.00	+0.21	+0.75^∗^	−0.50
1-Alkanols	−0.88^∗^	−0.53^∗^	−0.76^∗^	−0.51^∗^	−0.62^∗^	+0.18	+0.62^∗^
Alkanes	−0.80^∗^	−0.58^∗^	−0.50^∗^	−0.21	−0.22	+0.15	+0.60^∗^
1-Alkanol esters	−0.03	−0.06	−0.00	−0.00	+0.20	+0.08	+0.08
Glycol esters	−0.51^∗^	−0.18	−0.74^∗^	−0.01	−0.37	+0.16	+0.53^∗^
Phthalate esters	−0.19	−0.02	−0.21	−0.01	−0.22	−0.00	+0.15
Glycols	−0.15	−0.20	−0.10	−0.06	−0.07	+0.17	+0.03
β-Tocopherol	−0.01	−0.06	+0.00	−0.07	+0.00	+0.01	−0.01
Triterpenoids	−0.03	−0.06	+0.02	−0.31	−0.00	−0.27	−0.04
Steroids	−0.21	−0.22	−0.17	−0.04	−0.10	−0.01	+0.18
Caffeine	−0.13	−0.44	−0.09	−0.07	−0.00	+0.11	+0.45
Coverage	−0.56^∗^	−0.46	−0.28	−0.38	−0.18	+0.10	+0.56^∗^

Ad: adaxial; Ab: abaxial; Ew: epicuticular waxes; Iw: intracuticular waxes.

∗ *p* < 0.05.

Among the total leaf wax coverage (Ad + Ab), acids, 1-alkanols, alkanes, and glycol esters were found to be significantly and negatively correlated with the total leaf cuticular transpiration rates (T_Total_C_). Consistent with the total leaf wax coverage (Ad + Ab), these same waxes from the abaxial surface (Ab_Ew + Iw_) were also significantly and negatively correlated with the abaxial cuticular transpiration rates (T_Ab_C_); furthermore, these wax classes from the abaxial epicuticular layer (Ab_Ew_) were significantly and positively correlated with the abaxial epicuticular resistance (R_Ab/epi_C_). In abaxial intracuticular waxes (Ab_Iw_), acids and 1-alkanols were significantly and negatively correlated with the abaxial intracuticular transpiration rates (T_Ab/intra_C_).

Among the adaxial cuticular waxes (Ad_Ew + Iw_), 1-alkanols and alkanes were significantly and negatively correlated with the adaxial cuticular transpiration (T_Ad_). Aldehydes from the adaxial epicuticular waxes (Ad_Ew_) were significantly and positively correlated with the adaxial epicuticular resistance (R_Ad/epi_). In adaxial intracuticular waxes (Ad_Iw_), 1-alkanols were significantly and negatively correlated with the adaxial intracuticular transpiration rate (T_Ad/intra_; Table 1).

The correlations of cuticular transpiration rates or resistance with individual wax components from each cuticular substructure were also analyzed (Table 2). Seven wax components from the total wax coverage (Ad + Ab) were found to be significantly and negatively correlated with total leaf cuticular transpiration rates (T_Total_C_); they were two acids (C16 and C18), three 1-alkanols (C26, C28, and C32), and two alkanes (C25 and C29; Table 2). Four of them, namely, two acids (C16, C18) and two 1-alkanol (C28, C32), from the total abaxial wax coverage (Ab_Ew + Iw_) showed similar correlations with the abaxial cuticular transpiration rate. Such correlations were not found from the adaxial surface (Ad_Ew + Iw_). C25 alkane from the total leaf wax coverage showed a significant and negative correlation with the total leaf cuticular transpiration rate; such correlations were not found from the adaxial or the abaxial surfaces; instead, a significant and positive correlation was found on the abaxial epicuticular waxes between wax coverage and the abaxial epicuticular resistance (Table 2). In contrast, C29 alkane showed similar significant and negative correlations from the total leaf wax coverage (Ad + Ab), total adaxial wax coverage (Ad_Ew + Iw_), and total abaxial wax coverage (Ab_Ew + Iw_). C19 glycol ester and betulin from the abaxial surface (Ab_Ew + Iw_) showed significant and negative correlations with the abaxial cuticular transpiration rates; such correlations were not found from the total leaf wax coverage and the total adaxial wax coverage.

**Table 2:** Correlation analysis between cuticular transpiration rate (or resistance) and cuticular wax components (*R*^2^).

	Ad + Ab	Ad_Ew + Iw_	Ab_Ew + Iw_	Ad_Iw_	Ab_Iw_	Ad_Ew_	Ab_Ew_
C16 Acid	−0.58^∗^	−0.15	−0.78^∗^	−0.14	−0.64^∗^	+0.00	+0.53^∗^
C18 Acid	−0.60^∗^	−0.24	−0.74^∗^	−0.18	−0.69^∗^	+0.21	+0.49^∗^
C26 1-Alkanol	−0.80^∗^	−0.23	−0.30	−0.56^∗^	−0.40	−0.01	+0.03
C28 1-Alkanol	−0.78^∗^	−0.52	−0.59^∗^	−0.42	−0.60^∗^	+0.32	+0.51^∗^
C32 1-Alkanol	−0.64^∗^	−0.43	−0.65^∗^	−0.43	−0.39	+0.01	+0.25
C26 Aldehyde	−0.28	−0.34	ND	+0.00	ND	+0.59^∗^	ND
C28 Aldehyde	−0.42	−0.49	ND	−0.03	ND	+0.53^∗^	ND
C30 Aldehyde	−0.02	−0.31	+0.28	+0.06	+0.21	+0.83^∗^	−0.50
C21 Alkane	−0.18	−0.13	−0.31	−0.03	−0.07	+0.00	+0.55^∗^
C25 Alkane	−0.55^∗^	−0.41	−0.33	−0.10	−0.09	+0.35	+0.61^∗^
C29 Alkane	−0.85^∗^	−0.53^∗^	−0.64^∗^	−0.19	−0.22	+0.09	+0.50^∗^
C18 Glycol ester	−0.16	+0.00	−0.32	+0.06	−0.11	+0.20	+0.61^∗^
C19 Glycol ester	−0.40	−0.19	−0.69^∗^	−0.04	−0.43	+0.04	+0.35
C20 Glycol	−0.11	−0.29	−0.05	+0.00	−0.10	+0.59^*^	−0.00
Betulin	−0.36	−0.03	−0.52^∗^	−0.00	−0.39	ND	ND

Ad: adaxial; Ab: abaxial; Ew: epicuticular waxes; Iw: intracuticular waxes.

∗ *p* < 0.05.

On the adaxial surface, one wax component (C29 alkane) showed a significant and negative correlation with the adaxial cuticular transpiration rate; such correlations were not found from the adaxial epicuticular waxes or intracuticular waxes. Instead, another wax component (C26 1-alkanol) from the adaxial intracuticular waxes showed a significant and negative correlation with the adaxial intracuticular transpiration rate, although such correlation was not found from the total adaxial wax coverage. Four more wax components from the adaxial epicuticular waxes, namely, three aldehydes (C26, C28, and C30) and one glycol (C20), showed significant and positive correlations with the adaxial epicuticular resistance.

On the abaxial surface, seven wax components from the total abaxial coverage (Ab_Ew + Iw_) showed significant and negative correlations with the abaxial cuticular transpiration rates; they were two acids (C16 and C18), two 1-alkanols (C28 and C32), one alkane (C29), one glycol ester (C19), and one triterpenoid (betulin). Three of them (C16 acid, C18 acid, and C28 1-alkanol) from the abaxial intracuticular wax showed similar correlations with the abaxial intracuticular transpiration rates; a significant and positive correlation with the abaxial epicuticular resistance was also observed. The other three wax components (C32 1-alkanol, C19 glycol ester, and betulin) did not show correlations from neither the abaxial epicuticular waxes nor the intracuticular waxes.

The correlation analysis of the adaxial epicuticular waxes identified four more wax components showing significant and positive correlations with the adaxial epicuticular resistance (R_Ad/epi_), namely, three aldehydes (C26, C28, and C30) and one glycol (C20). These four components did not show correlations from neither the total adaxial waxes nor the adaxial intracuticular waxes. The correlation analysis from the abaxial epicuticular waxes identified seven wax components showing significant and positive correlations with the abaxial epicuticular resistance (R_Ab/epi_C_), namely, two acids (C16 and C18), one 1-alkanol (C28), three alkanes (C21, C25, and C29), and one glycol ester (C18). Four of them, namely, two acids (C16 and C18), one 1-alkanol (C28), and one alkane (C29), have been identified from the total abaxial waxes and the abaxial intracuticular waxes. The other three wax components (C21 alkane, C25 alkane, and C18 glycol ester) were found only from the abaxial epicuticular waxes to show such a significant and positive correlation.

## Discussion

The principal goal of this study was to quantify to which degree the epicuticular waxes and the intracuticular waxes contribute to the barrier against nonstomatal water loss in mature tea leaves. To address this question, cuticular transpiration rates from eight tea germplasms were measured from each leaf side and individual cuticular substructures. In addition, the epicuticular and intracuticular wax coverage and compositions from both leaf surfaces were determined.

### The Adaxial and the Abaxial Leaf Surfaces Showed Different Structures of Cuticular Transpiration Barrier

Cuticular transpiration takes place on both the adaxial and the abaxial leaf surfaces. The nonstomatal adaxial cuticle has been extensively studied, and it has been demonstrated that the adaxial intracuticular waxes constitute the major transpiration barrier, while the adaxial epicuticular waxes are not (Jetter and Riederer, 2016; Zeisler and Schreiber, 2016; Zeisler-Diehl et al., 2018). In contrast, there are few studies about the transpiration barriers from the abaxial leaf surface, largely due to the presence of stomata on this surface. Under normal growth conditions, stomatal transpiration is much higher than cuticular transpiration, which makes the measurement of the abaxial cuticular transpiration technically challenging. The recently established new method enables us to measure cuticular transpiration from different leaf surfaces and cuticular substructures simultaneously (Lino et al., 2020; Zhang et al., 2020). By applying this method, the cuticular transpiration rates from the eight tea germplasms were measured. On the adaxial surface, adaxial epicuticular resistance (R_Ad/epi_) was in the range of 10–25% (Figure 4B, upper panel); the total adaxial transpiration rate (T_Ad_) was significantly and positively correlated with the adaxial intracuticular transpiration rate (T_Ad/intra_; Figure 5). Therefore, our data in tea leaves were in accordance with previous conclusions (Jetter and Riederer, 2016; Zeisler and Schreiber, 2016; Zeisler-Diehl et al., 2018; Zhang et al., 2020). The cuticular transpiration rates from the abaxial surface were about 1.8–3.3 times higher than those of the adaxial surface (Figure 4). Thus, in addition to *Hedera helix* leaves (Šantrůček et al., 2004), tea leaves also showed higher abaxial cuticular transpiration compared with its adaxial surface. Correlation analysis showed that the total cuticular transpiration rate was significantly correlated with the abaxial cuticular transpiration rate rather than the adaxial cuticular transpiration rate (Figure 5).

The abaxial cuticular transpiration rate (T_Ab_C_) was positively correlated with the abaxial intracuticular transpiration (T_Ab/intra_C_) and negatively correlated with the abaxial epicuticular resistance (R_Ab/epi_C_). Our data suggest that the abaxial epicuticular waxes constituted another major transpiration barrier, while the abaxial intracuticular waxes were not (Figure 5).

It remains unclear why the adaxial and abaxial surfaces showed different structural organizations in the cuticular transpiration barrier, and what are the underlying mechanisms. Considering that the adaxial and the abaxial leaf surfaces have different niches such as light irradiance, insects, pathogen infestation patterns, etc., we speculate that the adaxial surface is optimized to restrict water loss, while the abaxial surface could be evolved to cope with other environmental stresses, which could compromise its potency as an efficient transpiration barrier. The different environmental niches could provide essential signals to differentially regulate wax biosynthesis or transport, which takes place within the adaxial epidermal cells and the abaxial epidermal cells, respectively. As a result, cuticle structural characteristics on the respective leaf surface could be affected. For crop breeding to improve drought tolerance traits or other related traits, more attention should be paid to germplasms with lower abaxial cuticular transpiration rates.

### Plant Growth Conditions Affected the Contribution of Structural Parameters to Tea Cuticular Transpiration Barriers

Most previous studies did not differentiate the epicuticular waxes and the intracuticular waxes, and bulk wax data were used to establish the cuticle structure–function relationship. Under this scenario, the important effects of substructures could be averaged out (Jetter and Riederer, 2016). This may explain the contradicting results from the literature regarding the cuticle thickness and wax coverage for the contributions to the cuticular transpiration barrier. Although no correlations have been reported between cuticular transpiration rate and cuticular wax coverage under normal growth conditions, the specific wax components, rather than wax coverage, have been found to affect cuticular transpiration (Riederer and Schneider, 2001; Jetter and Riederer, 2016). Water deficit induces the increase in cuticle thickness, which associates with the reduction in transpiration rate (Kosma et al., 2009; Jäger et al., 2014); similar observations were also found in tea following water deprivation treatments (Chen et al., 2020; Zhang et al., 2020). However, in other studies, cuticle thickness was not correlated with cuticular transpiration (Schreiber and Riederer, 1996; Jetter and Riederer, 2016; Bi et al., 2017). In this study, the eight germplasms were grown under normal conditions, cuticle thickness from the adaxial and the abaxial surfaces was measured, and no correlations were found between the cuticular transpiration rates and the cuticle thickness from both leaf surfaces. These data suggest that under normal growth conditions, cuticle thickness may not be a major structural parameter affecting the cuticular transpiration barrier.

However, the total leaf cuticular transpiration rates were found to be significantly and negatively correlated with the total leaf wax coverage (Table 1), which are consistent with the results from *Arabidopsis*, barley, tomato, and nectarine (Seo et al., 2011; Hasanuzzaman et al., 2017; Romero and Rose, 2019; Lino et al., 2020). In addition, the abaxial epicuticular resistance was significantly and positively correlated with the abaxial epicuticular wax coverage. Meanwhile, no significant correlations were found from individual leaf surfaces, neither the adaxial epicuticular and intracuticular waxes nor the abaxial intracuticular waxes (Table 1). Thus, the correlation between wax coverage and cuticular transpiration barrier could be species-specific or cuticle substructure-specific. This work highlights the importance of analyzing the wax coverage from cuticle substructure rather than using bulked data. Previously, we found that following drought treatment, the cuticular transpiration rates are significantly and negatively correlated with the adaxial and the abaxial intracuticular waxes; but no correlations were found between cuticular transpiration rates and the adaxial or the abaxial wax coverage (Zhang et al., 2020). These discrepancies suggest that wax coverage is not the only factor that affects the cuticular transpiration barrier. Instead, cuticle structural changes induced by other drought stress could play larger roles in affecting cuticular transpiration barrier properties. This notion was supported by the significant wax compositional changes following drought treatments (Chen et al., 2020; Zhang et al., 2020).

### Aliphatic Compounds and Glycol Esters Contributed to the Cuticular Transpiration Barrier in Tea Under Normal Growth Conditions

In this study, correlation analysis revealed that acids, 1-alkanols, alkanes, and glycol esters from the total leaf wax coverage (Ad + Ab) were associated with T_Total_C_ (Table 1). These data were in accordance with previous reports that the major cuticular transpiration barrier was formed by very-long-chain aliphatic compounds (Vogg et al., 2004; Jetter and Riederer, 2016). These same wax chemical groups (except acids and glycol esters) also showed similar roles on the adaxial and the abaxial surfaces as well as on the abaxial epicuticular waxes (Table 1). 1-Alkanols contributed to the transpiration barrier from the adaxial and the abaxial intracuticular waxes, while glycol esters specifically function on the abaxial epicuticular waxes layer (Table 1).

At the individual wax chemical level, there was no overlap of the contributing wax components between the adaxial and abaxial surfaces except C29 alkane, which contributed to the transpiration barrier on both leaf surfaces. The adaxial intracuticular waxes constituted a major leaf transpiration barrier, and 1-alkanol (C26) appeared to be the major contributor affecting its transpiration barrier properties. Although the adaxial epicuticular waxes were not a major transpiration barrier, three aldehydes (C26, C28, and C30) and one glycol (C20) were identified to affect its barrier properties (Table 2). The abaxial epicuticular waxes were another major transpiration barrier, and more wax components were identified as a contributor to their barrier formation (Table 2). Similar to the adaxial epicuticular waxes, the abaxial intracuticular waxes were not the major transpiration barrier. However, three wax components (C16 acid, C18 acid, and C28 1-alkanol) were identified to contribute to their transpiration barrier properties. These data suggest that for the individual wax component, its cuticular substructure localization affects its potency as a contributor to the transpiration barrier. This may explain the contradicting results from different plant species studied by different researchers (Riederer and Schneider, 2001; Seo et al., 2011; Jetter and Riederer, 2016; Hasanuzzaman et al., 2017; Romero and Rose, 2019; Lino et al., 2020). This also suggests that each cuticular compartment may have different microstructures, but a comparable transpiration barrier still can be formed.

The coverage of 1-alkanols and alkanes from the adaxial surface (Ad_Ew + Iw_) was higher than that of the abaxial surface (Ab_Ew + Iw_; Figure 3; Supplemental Data 1). Accordingly, compared with the abaxial surface, the adaxial surface showed a lower water transpiration rate and thus better barrier properties against water loss (Figure 4). On both leaf surfaces, 1-alkanols and alkanes were significantly and negatively correlated with the cuticular transpiration rate (Table 1). This suggests that differential distributions of specific aliphatic wax components on the adaxial and the abaxial surfaces could be one important factor to shape their barrier characteristics.

Generally, the adaxial leaf surface is densely covered with wax crystal, which deflects solar irradiance (Zhu et al., 2018; Zhang et al., 2020). 1-Alkanols and alkanes are the major components of wax crystal (Riederer and Schneider, 1990; Jetter et al., 2000; Cameron et al., 2006; Dragota and Riederer, 2007), which may explain why the adaxial epicuticular waxes were enriched with 1-alkanols and alkanes (Figure 3). Interestingly, both wax components did not contribute to the adaxial epicuticular resistance (Tables 1 and 2). Under field conditions, the adaxial surface is exposed to solar light directly; the deposition of wax compounds, especially 1-alkanols and alkanes, could increase the size and density of wax crystal and deflect solar radiation more efficiently, resulting in reduced leaf surface temperature and water loss (Long et al., 2003; Fukuda et al., 2008). However, the *in vitro* transpiration measurement in this study was performed under dark conditions; thus, it may miss out their true roles *in planta* under field conditions.

As a chemical group, triterpenoids did not show a significant correlation with the cuticular transpiration rate or resistance (Table 1), which is in accordance with previous studies (Jetter and Riederer, 2016; Staiger et al., 2019). However, at the individual chemical level, betulin, a triterpenoid from the abaxial surface, showed significant and negative correlations with the abaxial cuticular transpiration rate (Table 2). Previously, we found that triterpenoids play an important role in the formation of the cuticular transpiration barrier following drought stress (Zhang et al., 2020). The cuticular waxes from a desert plant are composed of mainly triterpenoids, which are deposited within the cutin matrix, and play a critical role in protecting the polymer against thermal expansion (Schuster et al., 2016). The studies of tomatoes and nectarines showed that triterpenoid deposition plays a major role in regulating fruit permeability during fruit development and water stress (Romero and Rose, 2019; Lino et al., 2020). Based on these findings, we speculate that triterpenoid deposition could modify cuticle transpiration barrier properties in response to stresses or specific developmental stages.

In summary, here, we conducted a comprehensive analysis of all cuticular substructures from eight tea germplasms. The adaxial intracuticular waxes and the abaxial epicuticular waxes constitute the major cuticular transpiration barriers; the abaxial cuticular transpiration rates were higher than the adaxial cuticular transpiration rates and significantly and positively correlated with the total leaf cuticular transpiration. The very-long-chain aliphatic compounds and glycol esters were important contributors to the overall cuticular transpiration barrier as well as in specific cuticle substructure of tea leaves. This work offered novel insights about the cuticular substructure–function relationship in tea plants.

## Data Availability Statement

The original contributions presented in the study are included in the article/Supplementary Material, further inquiries can be directed to the corresponding authors.

## Author Contributions

MC, CC, and YZ conceived the original research plans. MC and CC supervised the experiments. YZ, XK, ZD, HZ, ZY, and JQ performed the experiments. ZD provided technical assistance to YZ. MC and YZ designed the experiments and analyzed the data, and wrote the article with contributions from all the authors. MC agrees to serve as the author responsible for contact and ensures communication. All authors contributed to the article and approved the submitted version.

### Conflict of Interest

The authors declare that the research was conducted in the absence of any commercial or financial relationships that could be construed as a potential conflict of interest.
