# Supplementary material for: Leaf Cuticular Transpiration Barrier Organization in Tea Tree Under Normal Growth Conditions
Source: Front Plant Sci. 2021 Jun 30;12:655799. doi: 10.3389/fpls.2021.655799 (PMC8278822; doi:10.3389/fpls.2021.655799)
Supplement: Supplementary file 1 [file Table_1.docx]

| **Table S1.** Observed transpiration rates of the fifth leaf at 1^st^ h and 5^th^ h post excision under different treatments | | | | | | | |  |
| --- | --- | --- | --- | --- | --- | --- | --- | --- |
|  |  | **Control** | **+Ad/Vas** | **+Ab/Vas** | **+Ab/Vas::+Ad/Vas** | **-Ew_Ad_::+Ab/Vas** | **-Ew_Ab_::+Ad/Vas** | |
| ***Jinguanyin*** | 1 h | 0.156±0.014 | 0.135±0.015 | 0.068±0.006 | 0.043±0.004 | 0.073±0.006 | 0.305±0.18 | |
|  | 5 h | 0.123±0.011 | 0.108±0.016 | 0.059±0.008 | 0.032±0.003 | 0.070±0.012 | 0.267±0.016 | |
| ***0316B*** | 1 h | 0.158±0.013 | 0.133±0.010 | 0.064±0.005 | 0.041±0.003 | 0.074±0.006 | 0.310±0.019 | |
|  | 5 h | 0.126±0.009 | 0.110±0.013 | 0.057±0.004 | 0.031±0.004 | 0.063±0.010 | 0.259±0.013 | |
| ***Wuniuzao*** | 1 h | 0.165±0.007 | 0.138±0.014 | 0.074±0.004 | 0.043±0.005 | 0.088±0.009 | 0.298±0.016 | |
|  | 5 h | 0.132±0.010 | 0.107±0.009 | 0.062±0.007 | 0.036±0.005 | 0.071±0.013 | 0.248±0.018 | |
| ***0306A*** | 1 h | 0.205±0.009 | 0.180±0.012 | 0.094±0.008 | 0.055±0.003 | 0.098±0.008 | 0.352±0.019 | |
|  | 5 h | 0.162±0.014 | 0.156±0.016 | 0.078±0.006 | 0.043±0.005 | 0.085±0.008 | 0.307±0.015 | |
| ***0306H*** | 1 h | 0.193±0.014 | 0.171±0.013 | 0.086±0.005 | 0.047±0.004 | 0.094±0.006 | 0.344±0.012 | |
|  | 5 h | 0.157±0.010 | 0.141±0.014 | 0.069±0.003 | 0.037±0.003 | 0.073±0.015 | 0.292±0.009 | |
| ***Fuyun20*** | 1 h | 0.214±0.011 | 0.187±0.011 | 0.098±0.009 | 0.059±0.003 | 0.107±0.013 | 0.352±0.016 | |
|  | 5 h | 0.172±0.015 | 0.153±0.013 | 0.084±0.005 | 0.046±0.003 | 0.089±0.016 | 0.304±0.010 | |
| ***0202-10*** | 1 h | 0.257±0.011 | 0.229±0.015 | 0.109±0.010 | 0.066±0.007 | 0.114±0.009 | 0.379±0.014 | |
|  | 5 h | 0.206±0.016 | 0.186±0.010 | 0.080±0.005 | 0.053±0.004 | 0.087±0.011 | 0.326±0.017 | |
| ***Hongyafoshou*** | 1 h | 0.338±0.014 | 0.291±0.016 | 0.131±0.008 | 0.096±0.006 | 0.147±0.013 | 0.483±0.018 | |
|  | 5 h | 0.277±0.015 | 0.023±0.013 | 0.106±0.005 | 0.075±0.003 | 0.115±0.010 | 0.412±0.020 | |

Control: no gum arabic and vaseline treatment; +Ad/Vas: the adaxial leaf surface was sealed with vaseline; +Ab/Vas: the abaxial surface was sealed with vaseline; +Ab/Vas::+Ad/Vas: both leaf surfaces were sealed with vaseline; -Ew_Ad_::+Ab/Vas: the adaxial epicuticular waxes were removed by gum arabic while the abaxial surface was sealed with vaseline; -Ew_Ab_::+Ad/Vas: the abaxial epicuticular waxes were removed by gum arabic while the adaxial surface was sealed with Vaseline.
