# Supplementary material for: Leaf Cuticular Transpiration Barrier Organization in Tea Tree Under Normal Growth Conditions
Source: Front Plant Sci. 2021 Jun 30;12:655799. doi: 10.3389/fpls.2021.655799 (PMC8278822; doi:10.3389/fpls.2021.655799)
Supplement: Supplementary file 2 [file Presentation_1.PPTX]

## Slide 1
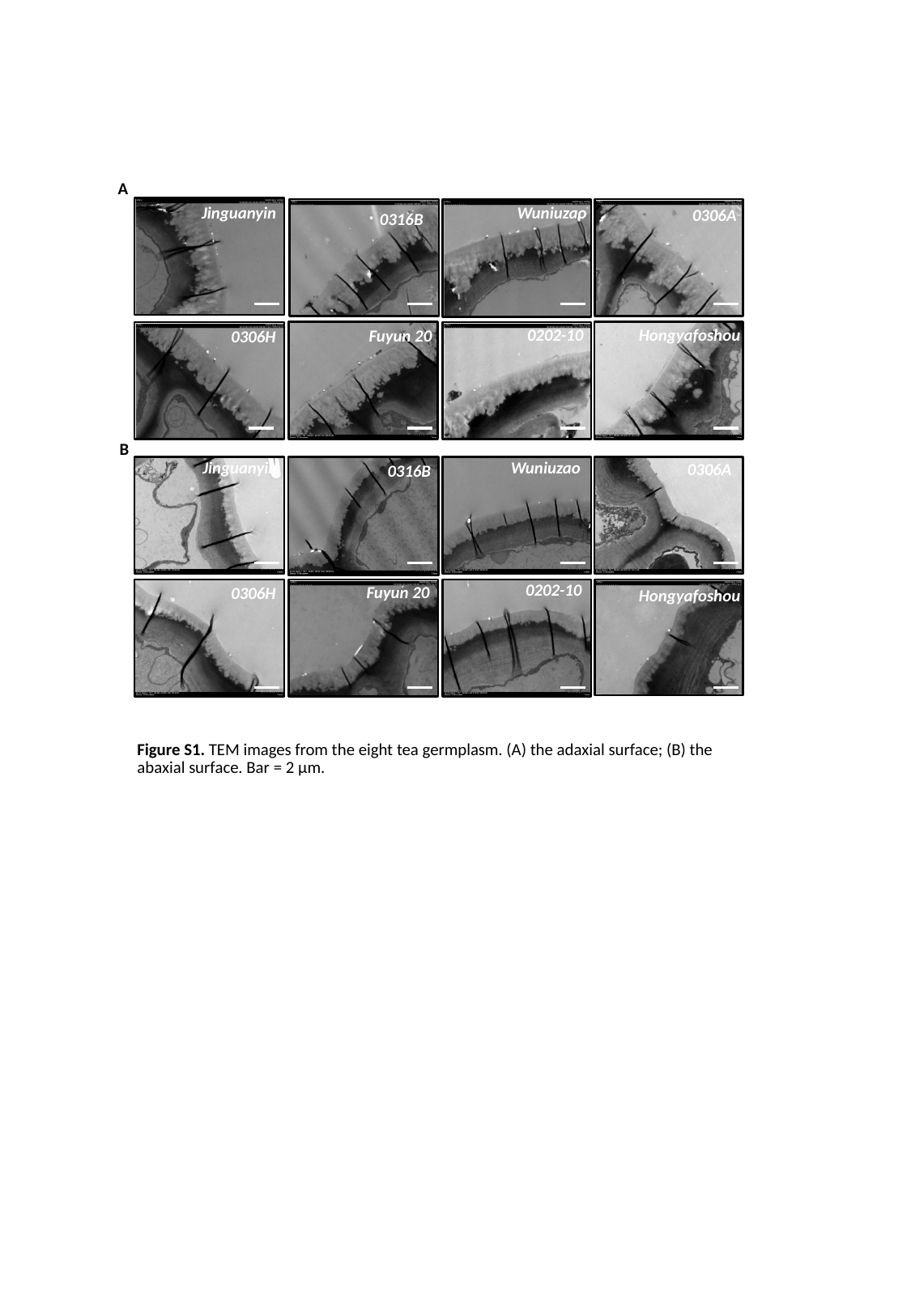

A
Wuniuzao
Jinguanyin
0306A
0316B
Hongyafoshou
0202-10
Fuyun 20
0306H
B
Wuniuzao
Jinguanyin
0306A
0316B
0202-10
Fuyun 20
0306H
Hongyafoshou
# Figure S1. TEM images from the eight tea germplasm. (A) the adaxial surface; (B) the abaxial surface. Bar = 2 µm.
