# Supplementary material for: Leaf Cuticular Transpiration Barrier Organization in Tea Tree Under Normal Growth Conditions
Source: Front Plant Sci. 2021 Jun 30;12:655799. doi: 10.3389/fpls.2021.655799 (PMC8278822; doi:10.3389/fpls.2021.655799)
Supplement: Supplementary file 3 [file Presentation_2.PPTX]

## Slide 1
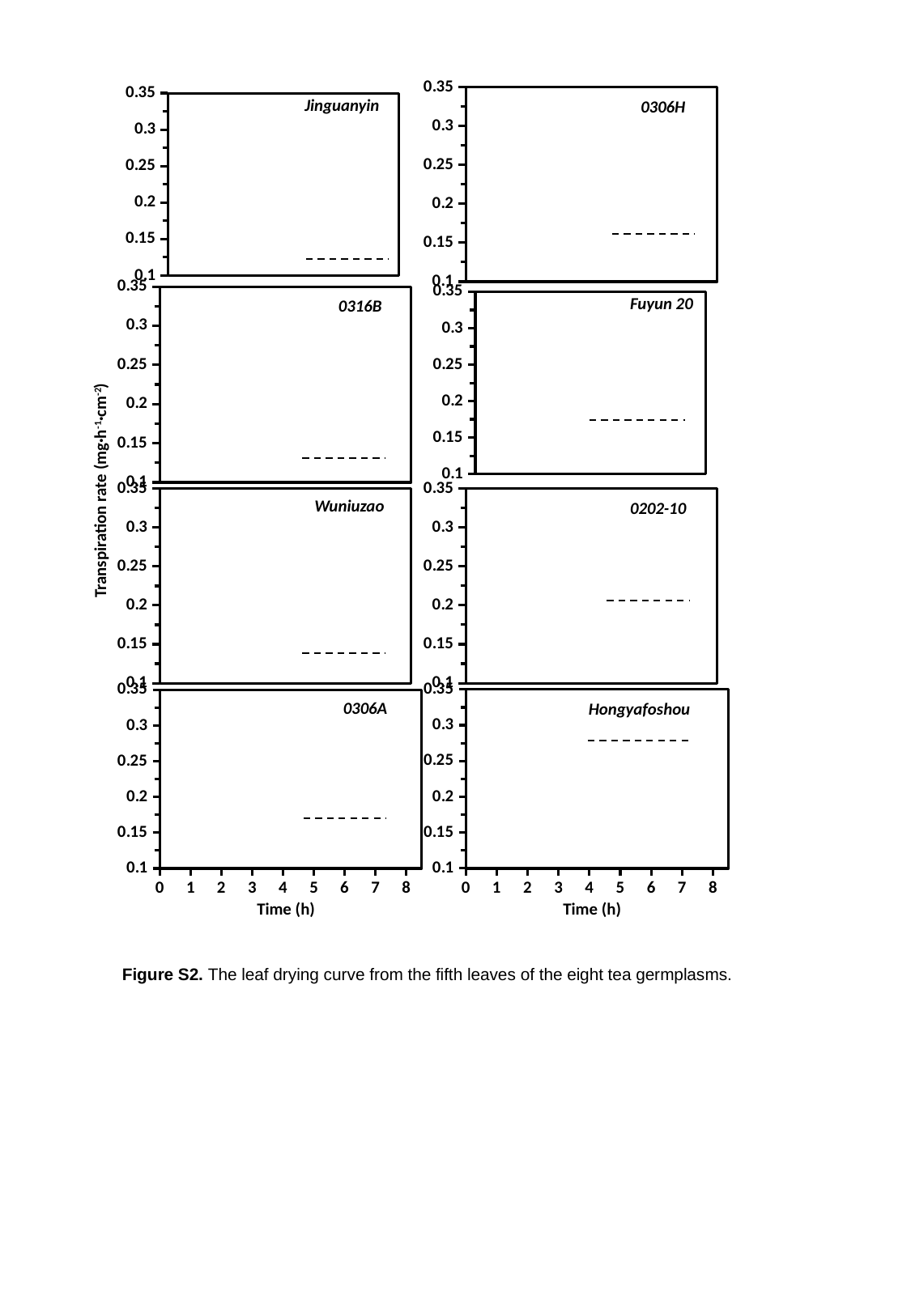

### Chart
| Category | TR |
|---|---|
### Chart
| Category | TR |
|---|---|Jinguanyin
0306H
### Chart
| Category | TR |
|---|---|
### Chart
| Category | TR |
|---|---|Fuyun 20
0316B
Transpiration rate (mg·h-1·cm-2)
### Chart
| Category | TR |
|---|---|
### Chart
| Category | TR |
|---|---|Wuniuzao
0202-10
### Chart
| Category | TR |
|---|---|
### Chart
| Category | TR |
|---|---|0306A
Hongyafoshou
Time (h)
Time (h)
# Figure S2. The leaf drying curve from the fifth leaves of the eight tea germplasms.
