# Supplementary material for: Leaf Cuticular Transpiration Barrier Organization in Tea Tree Under Normal Growth Conditions
Source: Front Plant Sci. 2021 Jun 30;12:655799. doi: 10.3389/fpls.2021.655799 (PMC8278822; doi:10.3389/fpls.2021.655799)
Supplement: Supplementary file 4 [file Presentation_3.PPTX]

## Slide 1
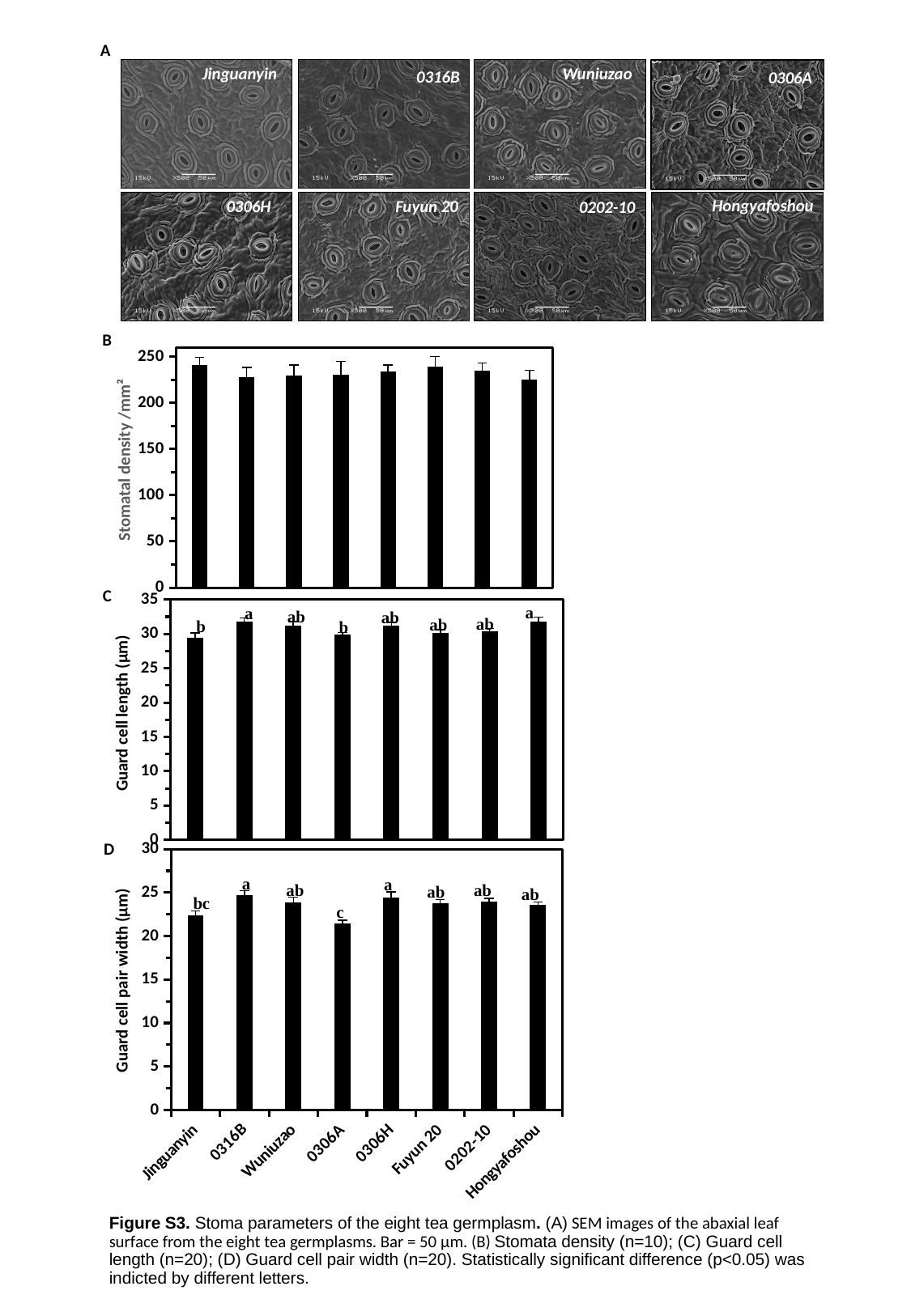

A
Jinguanyin
Wuniuzao
0316B
0306A
Hongyafoshou
Fuyun 20
0306H
0202-10
B
### Chart
| Category | Stomatal density /mm² |
|---|---|
| Jinguanyin | 240.0 |
| 0316B | 227.0 |
| Wuniuzao | 229.0 |
| 0306A | 230.0 |
| 0306H | 233.0 |
| Fuyun 20 | 239.0 |
| 0202-10 | 234.0 |
| Hongyafoshou | 225.0 |Stomatal density /mm²
C
### Chart
| Category | Guard cell length |
|---|---|
| Jinguanyin | 29.3967 |
| 0316B | 31.7697 |
| Wuniuzao | 31.129 |
| 0306A | 29.833999999999996 |
| 0306H | 31.128299999999996 |
| Fuyun 20 | 30.1239 |
| 0202-10 | 30.3354 |
| Hongyafoshou | 31.746699999999997 |a
a
ab
ab
ab
ab
b
b
Guard cell length (μm)
D
### Chart
| Category | Guard cell pair width |
|---|---|
| Jinguanyin | 22.368599999999997 |
| 0316B | 24.7024 |
| Wuniuzao | 23.820899999999998 |
| 0306A | 21.443 |
| 0306H | 24.391800000000003 |
| Fuyun 20 | 23.7115 |
| 0202-10 | 23.9075 |
| Hongyafoshou | 23.519099999999998 |a
a
ab
ab
ab
ab
bc
c
Guard cell pair width (μm)
Figure S3. Stoma parameters of the eight tea germplasm. (A) SEM images of the abaxial leaf surface from the eight tea germplasms. Bar = 50 µm. (B) Stomata density (n=10); (C) Guard cell length (n=20); (D) Guard cell pair width (n=20). Statistically significant difference (p<0.05) was indicted by different letters.
